# Stepping Into and Out of the Void: Funding Dynamics of Human Embryonic Stem Cell Research in California, Sweden, and South Korea

**DOI:** 10.1007/s12015-015-9626-4

**Published:** 2015-10-02

**Authors:** Noomi Weinryb, Tania Bubela

**Affiliations:** Department of Business Studies, Uppsala University, Box 513, Uppsala, Sweden 751 20; School of Public Health, University of Alberta, Edmonton, AB T6G 1C9 Canada

**Keywords:** Human embryonic stem cell, Research funding, Fundraising dependent nonprofit organization, Philanthropist, Accountability

## Abstract

Nonprofit organizations and philanthropists stepped into a funding void caused by controversies over public funding of human embryonic stem cell (hESC) research. Based on interviews of 83 representatives of 53 funders, we examine the motivations and accountability structures of public agencies, corporations, fundraising dependent nonprofit organizations and philanthropic organizations that funded hESC research in three jurisdictions: California, Sweden, and South Korea. While non-traditional forms of funding are essential in the early stages of research advancement, they are unreliable for the long timeframes necessary to advance cell therapies. Such funding sources may enter the field based on high expectations, but may exit just as rapidly based on disappointing rates of progress.

## Introduction

Controversies and debate over human embryonic stem cells (hESC) gave rise to novel funding models to support research on their therapeutic potential. A diversity of funders stepped into the void left by restrictions in the European Union and federal funding in the United States. Here we examine the funder motivations and their accountabilities to understand the complex funding environment in controversial research fields. Which funders stepped into the void to support hESC research, and why are some stepping out? To whom and how are these funders accountable? To what extent were jurisdictional differences in the blend of funding sources dependent on the level of political opposition to embryonic research and the cultural status of the embryo?

Opposition to hESC research stems from the moral status of the embryo - hESC derivation typically requires the destruction of early human embryos. Some groups grant embryos full moral status as a human being, others grant embryos a special status deserving of respect and protection based on potential, while others denounce the commodification of embryos as contrary to human dignity [1, 2]. Support for hESC research arises from the moral imperative to develop promising therapies for unmet medical needs [3] and the potential for economic development [4, 5]. Amidst continued controversy over hESC research in the United States [6] and renewed calls on the voters of California to support stem cell research and the development of regenerative medicine [7], it is timely to reflect on the motivations and dynamics of hESC funders over the past two decades.

The international hESC research policy environment is a patchwork of patchworks [8], which impacts funding priorities and the research conducted [9]. The environment is highly politicized - hESC research is one of few fields prohibited in some jurisdictions, with other notable examples being human germline modification [10] and human reproductive cloning [11]. Legal regimes that govern hESC may be broadly characterized as permissive - enabling the creation of embryos for hESC research; intermediate - placing restrictions on the derivation of embryos for hESC research; restrictive - prohibiting hESC research or derivation by limiting it to imported hESC lines; and jurisdictions that have no specific legislation in place (http://www.stemgen.org/stem-cell-world-map). HESC research in federated countries like the United States may be governed by an overlapping web of national and state laws, resulting in a mosaic of permissions and restrictions. Similarly, in the European Union a diverse legislative environment characterizes its member states.

Despite the policy environment, hESC research has progressed internationally. The International Stem Cell Registry lists 1304 hESC lines, of which 585 are available and 542 have data on provenance that enable researchers to determine compliance with local laws (http://www.umassmed.edu/iscr/). Publications on the lines vary from none to 743 (H9 (WA09) from the University of Wisconsin), with a median of one; only 51 lines have more than ten associated publications, while 207 have none. This skewed distribution reflects international restrictions that limit research to specific cell lines. Contrary to early expectations and this evidence of research intensity, clinical applications have been slow to eventuate - few clinical trials, primarily for forms of macular degeneration, are underway (Table 1) [12, 13].

**Table 1:** Clinical trials of hESCs or cells derived from hESCs registered in clinicaltrials.gov

Sponsor and Location^a^	Disease Targeted	Type of Cells derived from hESCs	NCT Identifier	Start Year	Phase
Geron/Asterias Biotherapeutics: CA, GA, IL	Spinal Cord Injury	oligodendrocyte precursor cells (GRNOPC1)	NCT01217008	2010	I
			NCT02302157	2014	I/II
Advanced Cell Technology/ Ocata Therapeutics: CA	Dry age-related and Stargardt’s macular dystrophy Myopic Macular Degeneration	retinal pigment epithelial cells (MA09-hRPE)	NCT01469832	2011	I/II
			NCT01345006	2011	I/II
			NCT01344993	2011	I/II
			NCT02463344	2015	Follow-up
			NCT02445612	2015	Follow-up
			NCT02122159	2014	I/II
CHABiotech: South Korea	Dry age-related macular degeneration	retinal pigment epithelial cells (MA09-hRPE)	NCT01625559	2012	I
			NCT01674829	2012	I/II
Pfizer: United Kingdom	Wet age-related macular degeneration	retinal pigment epithelial cells (PF-05206388)	NCT01691261	2012	I
Hôpitaux de Paris: France	Heart failure	cardiac-committed progenitor cells	NCT02057900	2013	I
Viacyte: CA, Canada	Diabetes Type 1	pancreatic endoderm cell in a pouch	NCT02239354	2014	I/II
Cell Cure Neurosciences: Israel	Dry age-related macular degeneration	retinal pigment epithelial cells (OpRegen)	NCT02286089	2014	I/II

^a^ *CA* California, *GA* Georgia, *IL* Illinois

Aside from political support or opposition to hESC research in different jurisdictions, culture and institutional environment additionally shape the organization of the research [14]. Increasingly, research relies on a mixture of funding sources [15] that are accountable in different ways to different sets of stakeholders. Accountability pressures may influence both the process for, and the substance of, funding decisions. For hESC research, funding sources include national/state public funding agencies; corporations; fundraising dependent nonprofit organizations that are primarily disease focused; and independently wealthy philanthropists. While the latter two categories may overlap with respect to legal and tax structures, we characterize philanthropists based on their freedom to donate their independent wealth to the public sphere. Philanthropists have actively shaped science policy, at least in the US, since the early 20th Century [16–18]. In Europe, philanthropists are increasingly active in the policy arena as state budgets shrink and the private sector plays an increasing role in the provision of public goods [19].

The concept of accountability is particularly important in contestable scientific fields, where funder influence on research and policy direction may result in these decision makers being questioned about their funding decisions. Accountability, or lack thereof, may influence the speed and ease with which funders enter and exit funding arenas. Accountability is relational between the funder and its external stakeholders. It may be upward to donors and governance boards, or downward to grantees [20–22]. It also gives rise to an obligation to explain and justify conduct; meaning external stakeholders may question and pass judgment on decisions, while funders may face the consequences of these decisions [23]. Accountability is the means by which “individuals and organizations are held externally to account for their actions and as the means by which they take internal responsibility for continuously shaping and scrutinizing organizational mission, goals, and performance” [24].

Differences in accountability arise between categories of funders and between countries. Public funding agencies are the most accountable because they exhibit all forms of accountability to the broadest set of stakeholders. Disease focused fundraising dependent nonprofits are, at a minimum, accountable to their governance boards, patient communities and donors [25]. Similarly, corporations are accountable to their boards, shareholders and clientele. Philanthropists, on the other hand, are not accountable to any specific funding constituency [26] but are at a minimum legally accountable [27].

Here we compare the motivations and accountability of public funding agencies, corporations, fundraising dependent nonprofits, and philanthropists in South Korea, Sweden, and the United States with a focus on California. These societies represent different regulatory and cultural environments. These jurisdictions harbor researchers who are active in the international hESC research community (Table 2), and their public funding agencies are among the core 11 members of the International Stem Cell Forum.

**Table 2:** Number of hESC lines and associated publications per jurisdiction from the International Stem Cell Registry (search August 2015)

Jurisdiction	Number of hESC lines	Number of associated publications
California	73	359
Korea	59	167
Sweden	68	298
Other	1104	3385
Total	1304	4209

## Background: hESC Research in the United States, Sweden, and South Korea

HESC has been most controversial in the United States where legal and political challengers have been at the forefront of funding debates. In the United States, controversy over hESC research dates to the seminal 1973 United States Supreme Court decision on abortion rights, *Roe v. Wade.* During the Reagan and Bush administration, funding was not available for research involving human embryos, which culminated in the 1995 Dickey-Wicker amendment that banned federal funding of human embryonic research [28]. The second Bush administration in 2001 went on to ban federal funding (primarily National Institutes of Health (NIH)) that had been allowed under the Clinton administration. The ban exempted research on the few hESC lines that had then been created. Some States, including California, stepped in with state funding initiatives to support hESC research. The California Institute for Regenerative Medicine (CIRM) was funded in 2004 from 3 Billion USD of State Bonds enabled through a plebiscite – Proposition 71 [29]. Despite ongoing legal disputes [30], the Obama administration lifted the Bush ban and enabled federal funding for research on hESC lines created after 2001. In contrast, hESC research has not been controversial in Sweden [31] even though opposition and regulatory restrictions exist at the level of the European Union [32, 33]. The liberal legislation regarding research on human embryos may be related to the barely contended status of abortions in Sweden, differing sharply from the US [34].

South Korea has had a unique trajectory of ethical contestation following the Hwang scandal [35]. Dr. Hwang Woo-Suk was a professor at the Seoul National University whose 2004 and 2005 *Science* publications on successful creation of hESCs by somatic cell nuclear transfer made him a national hero [36, 37]. Hwang later admitted ethical misconduct in obtaining ova from female graduate students and the black market [38] and his publications were found to report fraudulent data. Hwang was charged with embezzlement and ethical violations, was fired by Seoul National University, and his funding support was canceled. Hwang was eventually sentenced to a 2 year prison term for bioethics violations and embezzlement [39]. Hwang’s fall from grace affected Korea’s national psyche – the country had taken great pride in his international scientific stardom and his subsequent disgrace was reflected on the scientific community and its dreams of progress and economic advancement [40, 41].

## Methods

Our analysis is based on semi-structured interviews by N.W. between October 2011 and June 2012 with 83 representatives of 51 key funders in three jurisdictions: California, South Korea and Sweden (Table 3) and an additional 18 experts in hESC research for background information. We identified funders through internet searches, consultation with experts, and from acknowledgements in hESC research articles indexed in PubMed (Sweden October 2011, California January 2012, South Korea April 2012). The interviewees represented public funding agencies at state and national levels, corporations, nonprofit organizations, and independently wealthy philanthropists.

**Table 3:** Number of interviewees per jurisdiction and category of funder and number of funders represented by those interviewees

Type of Funder	United States No. Interviewees / No. Funders	Sweden No. Interviewees / No. Funders	South Korea No. Interviewees / No. Funders
Public Funding Agency	7 / 3	7 / 4	7 / 4
Corporation	6 / 4	10 / 5	3 / 2
Fundraising Dependent NonProfit	15 / 9	12 / 8	0 / 0
Philanthropist	6 / 5	9 / 6	1 / 1
Total	34 / 21	38 / 23	11 / 7

The interviews focused on the nature and structure of the funder, its motivations for funding hESC research, and its type and level of accountability to a variety of stakeholders. Using Excel, N.W. (1) read transcripts of the interviews, (2) inductively identified key themes, and (3) developed these into a code book with codes that identified and explained each theme. A trained Research Assistant then independently applied these codes. The coding was iterative, for example, when the research assistant identified two additional codes, N.W. re-analyzed the transcripts to incorporate these. This form of iterative coding by two independent coders is consistent with best practices in qualitative analysis, known as the constant comparison method [42, 43]. The inter-coder agreement was 91 % on the motivations and 95 % on the accountability data.

## Results

### Differences in Accountability of Funders of hESC Research

In our sample, all funders interviewed exhibited legal accountability. They adhered to regulations on the ethical conduct of research in general, and hESC research in particular. However differences arose between public funders and philanthropists as to the nature of their legal accountability. Public funders continuously monitored regulatory adherence: “*we have a team that actually audits projects and goes to the universities and companies on a regular basis to request information and demonstration that they’re keeping to our policies and meeting the standards we think that they should have and that they’ve agreed to under their funding arrangements*” (Interviewee 13, US Public Agency) and “*we go out and do compliance and education site visits and interactions with our funded investigators*” (Interviewee 15, US Public Agency)*.* Philanthropists, on the other hand, left legal accountability in the hands of their grantee institutions: “*It is entirely the university’s responsibility*” (Interviewee 61, Swedish Philanthropist) – “*it’s up to the university to enforce the specific rules and regulations*” (Interviewee 18, US Philanthropist).

Jurisdictional differences in accountability were also apparent. Swedish public funders and fundraising dependent nonprofits were more transparent in providing their financial information online than in California and South Korea. Swedish philanthropists also differed from those in other jurisdictions by demonstrating professional accountability in the form of membership in professional associations.

All categories of funders, except most philanthropists, demonstrated fiduciary accountability to their grantees through formal peer-review processes. Only Swedish philanthropists and one Californian philanthropist employed such a process. In explaining the lack of such processes, philanthropists emphasized their independence and tolerance for high risk research: “*what we looked for is science or paradigm shifting research and in that sense this is difficult to do when you have the scientific advisory committee that belongs to different schools of thought*” (Interviewee 21, US Philanthropist).

### Motivations for Stepping into and out of the Funding Void

Motivations for funding hESC varied according to jurisdiction and category of funder. Not surprisingly, corporations justified their funding based on potential profits or clinical promise, and public funders highlighted clinical promise and obligations to/directives from their political or bureaucratic masters. Fundraising dependent nonprofits emphasized a broader range of research goals, from basic research through clinical translation of therapies to benefit their constituent patient community.

#### United States

In the US, all but corporations cited supplemental funding as a justification for supporting hESC research. Restrictions on National Institutes of Health (NIH) funding for hESC research through the Bush Administration from 2001 to 2009 motivated disease focused fundraising dependent nonprofits to step into the void. *“*[Because the Bush policy left a regulatory vacuum for non-Federal sources of funds] *if you had private money you could do anything you wanted”* (Interviewee 19, US Philanthropist). Other philanthropists supported hESC research because they *“thought it was a very important cutting edge type of research for medicine”* (Interviewee 18, US Philanthropist). Some fundraising dependent nonprofits were specifically founded to support the research, including two which ran independent laboratories with non-federal funds. There was a *“sense of urgency to move forward (…) we were the stop gap, we made sure that things made it through the lean years and we’re primed and ready for huge advances”* (Interviewee 10, US Nonprofit).

Because the Bush funding ban left not only a resource vacuum, but also a policy vacuum, philanthropists and nonprofits also supported the development of policies and guidelines *“if we didn’t do it, nobody was going to do it”* (Interviewee 19, US Philanthropist). US philanthropists stood out from other funders in citing personal reasons, for example, direct donations to the research program of a clinician who successfully treated a family member. The motivations of philanthropists, therefore, highlighted their freedom and independence in two ways. First, they remedied a gap in public policy, and second, they donated based on personal relationships, without application of scientific norms, such as peer-review.

Among the US funders, interviewees from fundraising dependent nonprofits struggled to manage the expectations of their donors and/or disease communities for hESC research. In balancing their advocacy roles with their accountability to these external stakeholders, nonprofits felt their role was to: “*diffuse the hype (…) to educate, not only by our funding, but by the things that we talk about in public forums*” (Interviewee 12, US Nonprofit) because “*patients sort of see it as the ultimate cure, the ultimate treatment option*” (Interviewee 25, US Nonprofit); *“so, we’re very, very conservative with reporting progress”* (Interviewee 29, US Nonprofit). However, conservative messaging was not well received because: “*I don’t know that people like to hear this sort of cautionary side about ESC*” (Interviewee 12, US Nonprofit). As stated by the same interviewee: “*A lot of the stuff we see is either so basic and is at such a remove from* [the condition] *that it should be more appropriately funded by the* [public funder] *(…) or it is for* [the condition] *but it’s just really lousy science. (…) the field had gotten way ahead of itself, that there was too much myth and hype, and still even today, there’s so much we don’t know about* [hESC] (Interviewee 12, US Nonprofit). Others feel that they funded initially to launch the field, but since the entry of public funders, they have “*kind of scaled back”* (Interviewee 23, US Nonprofit). “*Frankly, our money in an environment like that wasn’t really making as big an impact as having state money go to it. So we kind of backed off (…) Maybe SC will never be the cure-all that we want it to be. (…) Who knows for sure, but the real cure will come from some other kind of treatment* (Interviewee 25, US Nonprofit).

#### Sweden

Representatives of Swedish public funders were uncertain of the extent to which they supported hESC research or why they had funded it. These funders assessed hESC research alongside other types of research without applying any special considerations - they emphasized scientific progress and economic growth. These justifications were echoed by all types of funders: *“Scientific quality is the only criterion of evaluation”* (Interviewee 61, Swedish Philanthropist). In Sweden, differences between public and private funders were less apparent, and private funders similarly adhered to scientific norms for accountability, such as peer-review.

However, sounding a cautionary note, Swedish fundraising dependent non-profits also commented on the hype surrounding hESC research. While the field started with a lot of optimism, it has become: *“more realistic about the whole thing and it takes longer, it is not as easy as you thought (…) and maybe the focus has shifted from embryonic cells.”* (Interviewee 56, Swedish Nonprofit). Indeed, *“nowadays maybe embryonic, it’s a little bit out of fashion (…) it hasn’t been that successful so far”* (Interviewee 59, Swedish Nonprofit).

#### South Korea

In South Korea, both private and public funders emphasized the therapeutic potential of hESCs compared to other cell types, global research leaders, the size and profile of the rare diseases community, the “*strong representation for IVF* [in vitro fertilization]” (Interviewee 38, Korean Corporation) and supportive policies for the derivation of novel hESC lines, all of which contributed to prioritizing hESC research as a field in which Korea could be globally competitive. Funders indicated growing competition with China as a major driver and the need to catch up to the same level of funding and research as the US and UK. South Korean funders were the most similar to each other in motivation and accountability structures.

Korean funders also reflected on the damage of the Hwang scandal: “*Dr. Hwang gave the public and overestimated blue print to commercialization*” (Interviewee 41, Korean Public Agency) and “*We had to deal with our reputation*” (Interviewee 38, Korean Corporation). Interviewees used terms such as “*Trauma*”. The damage to public trust has longer-term implications, with the public “*bored*” and “*fed up*” with the issue: “*but still, our general public has some feeling against Stem Cell discoveries and unfortunately that doubt extended to other basic research (…) so if some researchers say wow, this is first in the world (…) they say oh is it really true or another* [Hwang scandal]” (Interviewee 35, Korean Public Agency).*”* Nevertheless, Korean funders also felt that the scandal had served a public relations purpose with the Korean public and motivated additional funding through a special presidential drive in 2011/2012. “*His* [Hwang’s] *research output made a big issue in Korea with like the stem cell researchers. So, about stem cells, the Korean people, this made people to know about stem cells. So, when people, they’re interested in stem cell research also and there’s some issues makes like that, budget for the research is bigger on some issues*” (Interviewee 42, Korean Public Agency).

## Discussion

HESC research has attracted a range of funders, internationally. Their motivations and accountabilities varied in the three jurisdictions examined here. Public funders, as the most accountable, are tied to the policies of their political masters and strict financial and fiduciary obligations to tax payers and grantees. This manifests through formal processes of peer review and financial reporting. While subject to external political forces, these agencies are the least nimble in responding to changing research environments. Corporations by contrast are primarily driven by clinical promise and profit motives, and therefore may rapidly enter and exit research fields.

In addition to public and corporate research funding, hESC has also benefited from support from fundraising dependent nonprofits, often disease focused, and philanthropists. While fundraising dependent nonprofits were highly responsive to their patient constituencies in funding the research and in supporting enabling policy, our study shows that some are currently re-examining their support for the field because, contrary to patient and funder expectations for the potential therapeutic benefits of hESCs [44], clinical trials have been slow to materialize [12, 13]. No therapies have yet received regulatory approval and may face hurdles in achieving market acceptance both for ethical and for cost considerations; health care systems and private payers are increasingly concerned with the cost of novel therapies.

Because fundraising dependent nonprofits are socially accountable to their donors and disease communities, they are also highly responsive to research trends. Philanthropists are even less accountable, and their funding is largely premised on personal relationships and the interests of individual philanthropists. While these forms of funding are essential in the early stages of research advancement, they are unreliable for the long timeframes necessary to advance cell therapies. Such funding sources may enter the field based on high expectations, but may exit just as rapidly based on disappointing rates of progress. For the development of therapies premised on understanding of complex biological systems, public funding sources are the most stable, but may be equally unreliable where that research is ethically controversial. HESC research, especially in North America, advanced in funding and policy only because of the participation of fundraising dependent nonprofits and philanthropists. Retaining the trust of those funders and the constituencies they represent is paramount. Our study shows that locally contingent interpretations of the research may influence the funding process and, in this sense, circumvent or influence traditional accountabilities built into the peer review system, altering motivations and directions of large amounts of scientific funding.
